# A Case Report of Primary Hyperoxaluria Type 1 Diagnosed in a Geriatric Patient Receiving Dialysis: Unique Clinical Course With Lumasiran

**DOI:** 10.1016/j.xkme.2026.101503

**Published:** 2026-08-13

**Authors:** Matthew C. Breeggemann, Marshall L. Stoller, Stephen L. Gluck

**Affiliations:** 1 Division of Nephrology, Department of Medicine, University of California San Francisco, CA; 2 Department of Urology, University of California San Francisco, CA

## Abstract

Primary hyperoxaluria type 1 (PH1) is a rare inherited disorder that typically presents in childhood and often progresses to kidney failure. We report an 83-year-old woman with kidney failure of unknown etiology, recurrent calcium oxalate nephrolithiasis beginning at age 50, and a family history of nephrolithiasis and kidney failure. After starting hemodialysis, evaluation revealed marked hyperoxaluria (194 mg/d), elevated plasma oxalate (34 μmol/L), and homozygous AGXT p.Pro11Arg mutations consistent with PH1. Treatment with pyridoxine and lumasiran was initiated. Five months later, approximately one year after dialysis initiation, the patient discontinued hemodialysis because of post-dialysis fatigue. During 12 months of follow-up, dialysis was not required, creatinine clearance remained 19 mL/min, plasma oxalate normalized or remained near normal, and stone burden remained stable. To our knowledge, this is the first reported non-transplanted PH1 patient to successfully discontinue hemodialysis following lumasiran therapy, highlighting the potential benefit of RNA interference therapy even in advanced disease.

## Introduction

Primary hyperoxaluria (PH) is a group of rare autosomal recessive disorders of impaired hepatic glyoxylate metabolism. PH type 1 (PH1), the most common and severe phenotype, results in incident kidney failure in most patients by young adulthood.^1^ The majority of patients with PH1 are diagnosed around age 10 years, although a significant delay in diagnosis is common.^2^ Preventative treatment options have historically been limited to robust fluid intake, crystallization inhibitors, and pyridoxine. The newest approach for PH1 management involves RNA interference (RNAi) therapies, lumasiran or nedosiran, which reduce urine oxalate and plasma oxalate (POx) levels. Hemodialysis (HD) is implemented to reduce complications from oxalosis in those with advanced chronic kidney disease. These strategies are used as a bridge to combined liver-kidney transplantation, which is curative. We present a highly unusual case of PH1 featuring a patient with advanced age at diagnosis and the only known instance of a nontransplanted patient receiving dialysis successfully stopping HD after RNAi therapy initiation.

## Case Presentation

An 83-year-old woman with kidney failure of unknown etiology and recurrent calcium oxalate nephrolithiasis presented for care. She first began passing kidney stones around age 50 years and has required 6 stone removal surgeries. She completed a 24-hour urine collection 5 years prior to presentation that was notable for an elevated urine oxalate of 194 mg/d (reference <40 mg/d). Her family history included 2 deceased siblings who also had kidney failure of unknown etiology and recurrent nephrolithiasis. She started thrice weekly in-center HD 1 month prior to presentation for uremia. Imaging was notable for large bilateral nonobstructing stones measuring up to 1.6 cm. She did not have radiographic evidence of nephrocalcinosis. A predialysis POx level was elevated at 34 μmol/L (reference ≤2.0 μmol/L). Genetic testing was notable for homozygous mutations (p.Pro11Arg) in the *AGXT* gene, consistent with a diagnosis of PH1. She was started on pyridoxine and lumasiran. Approximately 12 months after HD initiation and 5 months after pyridoxine/lumasiran initiation, the patient stopped presenting for HD treatments due to postdialysis fatigue. There have been no indications for dialysis reinitiation over the subsequent 12 months. She has adequate kidney function (creatinine clearance 19 mL/min) and consistently normal or near-normal POx levels (Fig 1). The stone burden remains stable on imaging.

**Figure 1:**
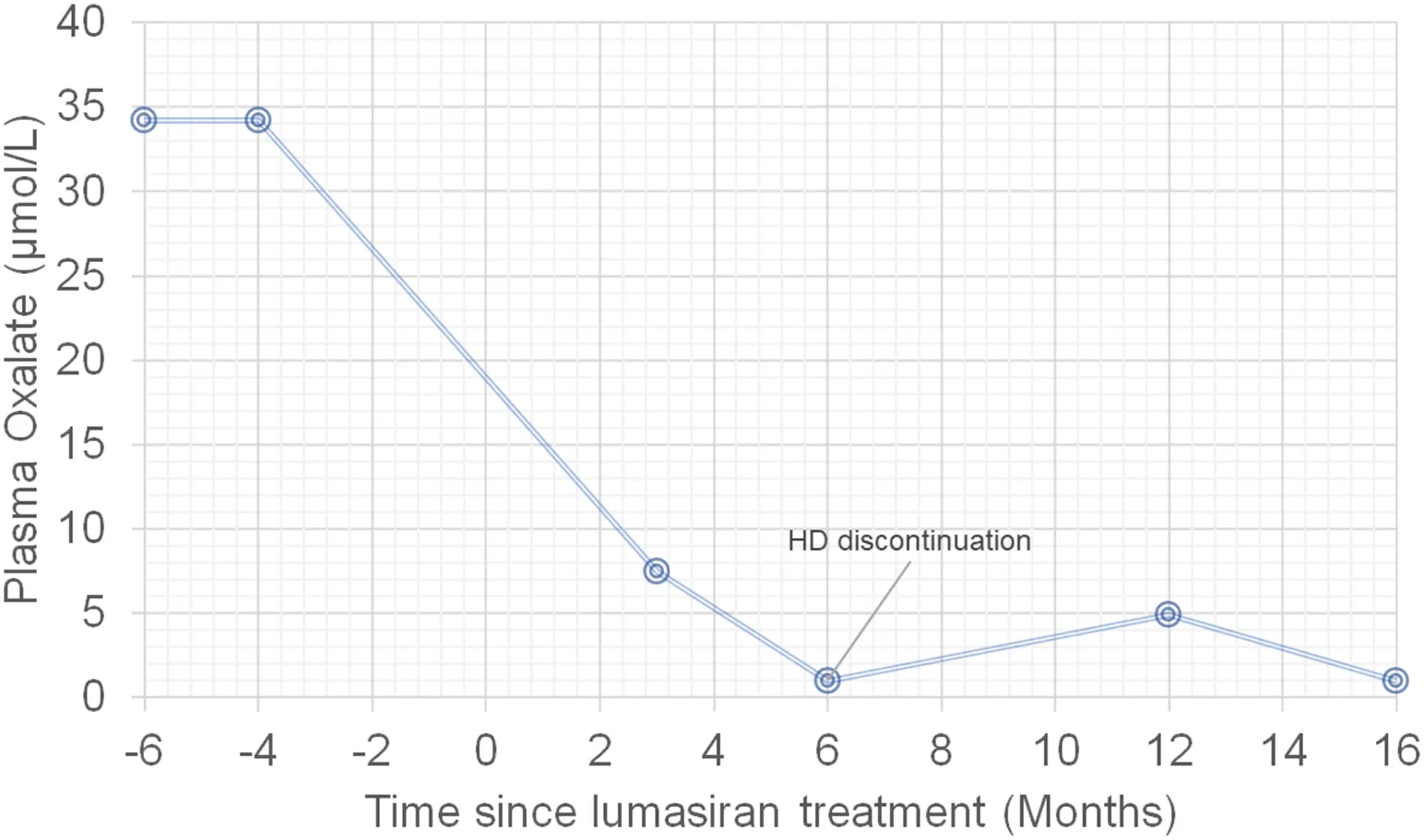
Change in plasma oxalate (μmol/L). HD, hemodialysis.

## Discussion

PH1 is a devasting metabolic disease resulting from the hepatic overproduction of oxalate. PH1 patients suffer from frequent stone episodes and are at risk for oxalosis and advanced chronic kidney disease requiring HD and transplantation. This outlook has improved with RNAi therapies that are effective at reducing urine oxalate and POx levels in patients who fail to adequately respond to pyridoxine. For those with advanced chronic kidney disease, POx becomes the surrogate endpoint because urine oxalate is no longer a reliable marker of disease activity.^3^ Genetic testing, when available, is used to evaluate for possible PH,^4^ and gene editing trials are currently being conducted as potential additional treatment options for PH1 patients (NCT06839235, NCT06892301). Our case highlights the importance of maintaining a high index of suspicion for patients of any age who may have PH1 to minimize diagnostic delays and provide prompt disease-altering therapies. Although initially against medical advice, our case represents the first known nontransplanted PH1 patient who successfully weaned off of HD after starting lumasiran. We speculate that she may have had undetected nephrocalcinosis and experienced renal recovery after RNAi initiation. It is important to note that our patient also continued pyridoxine, despite not having a vitamin B_6_-responsive *AGXT* mutation, given the low risk with this intervention. Vitamin B_6_ serves as a cofactor and increases the efficacy of the alanine-glyoxylate aminotransferase enzyme in some situations.^5^, ^6^, ^7^ We were unable to determine what effect, if any, pyridoxine had for our patient as it was continued alongside lumasiran.

In conclusion, many unanswered questions remain for optimal management of PH1, including the potential for gene editing approaches and the ideal transplantation strategy for patients with advanced chronic kidney disease who are on RNAi therapy. There is also an urgent need for effective treatments for those with PH types 2 and 3.
